# Utilization of natural deep eutectic solvents and ultrasound-assisted extraction as green extraction technique for the recovery of bioactive compounds from date palm (*Phoenix dactylifera L.*) seeds: An investigation into optimization of process parameters

**DOI:** 10.1016/j.ultsonch.2022.106233

**Published:** 2022-11-21

**Authors:** Jennifer Osamede Airouyuwa, Hussein Mostafa, Asad Riaz, Sajid Maqsood

**Affiliations:** a Department of Food Science, College of Agriculture and Veterinary Medicine, United Arab Emirates University, Al-Ain 15551, United Arab Emirates; b Water and Energy Center, United Arab Emirates University, Al-Ain 15551, United Arab Emirates

**Keywords:** Green extraction, Ultrasound-assisted extraction, Natural deep eutectic solvents, Date seeds, Phenolic compounds, Antioxidant activity

## Abstract

The green extraction of bioactive compounds from date seeds was investigated using seven natural deep eutectic solvents (NADES) coupled with ultrasound-assisted extraction (UAE). The seven NADESs mainly consisted of choline chloride as hydrogen bond acceptors (HBA) and four sugars, two organic acids, and one polyalcohol as hydrogen bond donors (HBD) were utilized in this study. When the extraction efficiency of the NADESs was compared to that of the conventional solvents, all the NADESs showed superior bioactive compounds recovery efficacy. The lactic acid-based NADES had the highest extraction efficiency and was further optimized using the response surface method and Box-Behnken design. A four-factors including extraction time (10, 20, and 30 min), ultrasound amplitude (70, 80, and 90 %), % NADES content (30 %, 50 %, and 70 %) and solid-to-solvent ratio (1:30, 1.5:30, and 2:30 g/ml) each at three levels (−1, 0 and 1) using Box-Behnken design was applied. The % NADES content and the solid-to-solvent ratio were the major factors influencing the extraction efficiency of the total phenolic content (TPC) and the 1,1-diphenyl-2-picrylhydrazyl (DPPH) radical scavenging activity. The optimum extraction conditions included an extraction time of 15 min, ultrasound amplitude of 90 %, % NADES content of 70 % and solid-to-liquid ratio of 1:30 g/ml. The experimental values for TPC and DPPH at optimum extraction conditions were 145.54 ± 1.54 (mg GAE/g powder) and 719.19 ± 2.09 (mmol TE/g powder), respectively. The major phenolic compounds observed in the date seeds extracted using ChCl-LA were 3,4-dihydroxybenzoic acid, catechin and caffeic acid. This study reveals that the extraction of date seeds with NADES in combination with UAE technique was able to recover significantly higher amounts of phenolic compounds which could find useful applications in the food, pharmaceutical, and cosmetics industries.

## Highlights

• Seven NADES for the extraction of bioactive compounds from date seeds were synthesized.
• Innovative extraction techniques “ultrasound-assisted extraction” coupled with NADES were employed in the extraction of date seeds.
• All the NADES had better extraction efficiency for phenolic compounds than conventional solvents.
• The NADES with the better physicochemical property was optimized to enhance phenolic content and antioxidant activities.
• 3,4-Dihydroxy benzoic acid, catechin, and caffeic acid were the main phenolic compounds present in date seeds.

## Graphical abstract

**Figure:**
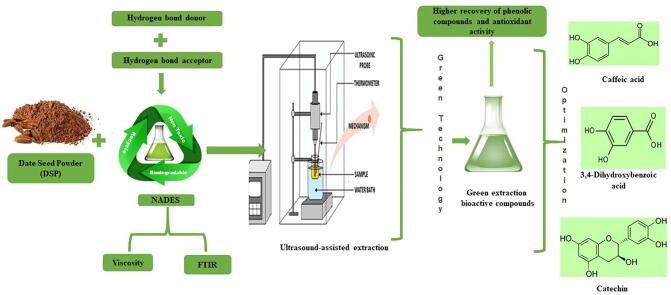

## 1 Introduction

Date fruit are commonly cultivated in the middle east and considered the most popular consumed staple food in this region since it is rich in nutritional [1] and possess diverse medicinal properties, such as hypoglycemic, antioxidant, antimicrobial, and anticancer activities [2]. Date palm tree and fruit constitute a large amount of waste which may constitute environmental challenges if not properly handled, these wastes include spikelets, seeds, and leaves. Studies have shown that these waste products from dates such as the seeds contain ample amounts of fiber and a considerable amount of proteins, vitamins, and lipids [3], [4]. The date seeds are also rich in bioactive compounds (flavonoids and polyphenolic compounds) [5], [6]. These bioactive compounds possess potential health attributes such as anti-inflammatory actions, anti-mutagenic, antioxidant, and anti-carcinogenic activities [7].

In the past decades, the use of conventional processes of extraction has been exploited for the recovery of bioactive compounds from plant sources. However, these conventional processes have considerable drawbacks in terms of extraction solvents used which are not sustainable as it requires a high volume of solvents which makes the process costly, low yield as a result of thermal degradation, and they are flammable and hazardous with limited disposal and recycling possibilities [8]. To achieve a sustainable process of extracting bioactive compounds, there is an increasing need to employ the green extraction method which are considered to be safe and sustainable. Green extraction is a recent goal in scientific and industrial research development, as it is designed to reduce or eliminate the utilization of toxic chemical solvents, reduce energy consumption with minimal environmental impact and a huge benefit to human health [9], [10], [11]. The alternative to conventional solvents are green solvent among which the Ionic Liquid (IL) are first to be used as green solvents. The benefits of IL over conventional solvents are their low vapor pressure, a wide range of miscibility and solubility, good thermal characteristics, and good recyclability [12]. However, ILs do possess several disadvantages as well, for example their high cost of production, toxicity, and low biodegradability [13], [14]. The use of deep eutectic solvents (DES) has gained more attention as an ecofriendly green solvent and a good alternative to conventional solvents. Deep eutectic solvents are synthesized by mixing non-ionic compounds, usually, salts and molecular compounds where one of the components acts as a hydrogen bond donor (HBD) and the other as a hydrogen bond acceptor (HBA) at a defined molar ratio [15]. Recently, DES produced from primary metabolites, and or bio-renewable materials are called natural deep eutectic solvents (NADES) [16]. The application of DES/NADES in the extraction of bioactive compounds from natural sources and the synthesis of different classes of DES have been widely investigated [17], [18], [19], [20], [21]. In a recent study by Kehili, Isci, Thieme, Kaltschmitt, Zetzl and Smirnova [22] reported a higher polyphenol yield of 128 g GAE/kg dry biomass from Deglet Nour variety of defatted date seeds extracted with DES coupled with microwave assisted extraction. Similarly, DES/NADES has been efficiently utilized for the extraction of biomolecules from plant sources such as phenolic compounds [22], [23], [24], [25], flavonoids, anthocyanin [16], [26], [27], [28] protein [29], [30], [31], [32] and polysaccharide [33].

Innovative extraction techniques like microwave-assisted and ultrasound-assisted extraction (UAE) are eco-friendly and economical. The combination of innovative extraction techniques and green solvents results in better recovery of bioactive compounds when compared with conventional methods [34]. The ultrasound-assisted extraction technique, generates acoustic cavitation, and thermal and mechanical effects produced by the frequency of the ultrasonic waves caused by the expansion and contraction of material to be treated helps in effective extraction of bioactive compounds [35], [36], [37]. The UAE has an advantage over the conventional extraction method due to its short time of extraction, little amount of extraction solvent required, and high extraction yield [38].

To reduce the waste generated from date palm fruit, the date seeds are potentially rich bioresource which can be used to recover the bioactive compounds with immense benefits to human health and wellbeing. Most of the studies conducted on recovery of bioactive compounds from date seeds have been carried out using conventional solvents. Using green extraction solvent (NADES) are most feasible and safe solvent as the bioactive compounds recovered can be readily used in food formulation after confirming their toxicity status. Therefore, this study aimed at employing green solvents in combination with emerging extraction techniques to effectively recover the bioactive compounds from the date seeds. The ecofriendly method utilized was the UAE for the optimal recovery of total phenolic compounds using NADES as green solvents. To optimize this process, response surface methods (RSM) were employed and the optimization conditions were solid to solvent ratio, extraction time, ultrasound amplitude, and the percentage NADES content. Furthermore, the physicochemical characteristics of the synthesized NADES such as viscosity, and FTIR were also investigated.

## 2 Material and methods

### 2.1 Material

Khalas variety of date seeds at the matured stage (Tmar) was obtained from Al FOAH Date Company, Abu Dhabi, UAE. The NADES reagents were purchased from Sigma-Aldrich (St. Louis, MO, U.S.A.). Gallic acid, sodium acetate, 6-hydroxy-2,5,7,8-tetramethylchroman-2-carboxylic acid (Trolox), rutin (quercetin 3-O-rutinoside), 2,2-diphenyl-1-picrylhydrazyl (DPPH), Folin-Ciocalteu reagent, and 2,2′-azino-dis [3-ethylbenzthiazoline sulfonate (ABTS) were from Sigma-Aldrich (St. Louis, MO, U.S.A.). The other reagents not mentioned were analytical grades procured from Fisher Scientific (Nepean, ON).

### 2.2 Date seed sample preparation

The date seeds (khalas variety) were washed with distilled water to remove any adhered date fruit pulp. Seeds were then dried in a hot air oven at a temperature of 40 °C and ground using an industrial seed grinder at Al FOAH experimental farm in Abu Dhabi. To obtain a fine date seed powder, the ground seeds were further pulverized using a sieve of <200 µm, the date seeds powder was stored at 4 ℃ for further analysis.

### 2.3 Preparation of NADES

The NADES were prepared by the heat and stirring method as described by Shang et al. [39]. Seven different NADESs were synthesized (Table 1) using choline chloride as hydrogen bond acceptor (HBA) and the hydrogen bond donor (HBD) comprises two carboxylic acid-based (lactic acids and malic acids), four sugar-based (glucose, sucrose, xylose, and xylitol) and a polyalcohol-based (1,4-butanediol) NADES.

**Table 1:** NADES abbreviations, constituents, and viscosity (cP) at different temperatures.

Solvent abbreviation	NADEs Composition			Molar ratio	Viscosity (cP) at 40 °C	Viscosity (cP) at 60 °C
	Component 1	Component 2	Component 3			
ChCl-Glu	Choline Chloride	Glucose	Water	1:1:0.5	1360	990
ChCl-Suc	Choline Chloride	Sucrose	Water	1:2: 0.5	7000	2100
ChCl-Xyli	Choline Chloride	Xylitol	Water	1:1:0.5	770	440
ChCl-Xylo	Choline Chloride	Xylose	Water	1:1:0.5	920	380
ChCl-LA	Choline Chloride	Lactic acid		1:2	20	10
ChCl-MA	Choline Chloride	Malic acid		1:1	1710	480
ChCl-But	Choline Chloride	1,4-Butanediol		1: 3	10	10
cP* Centipoise						

### 2.4 Ultrasound-Assisted extraction (UAE) of date seed powder

To extract bioactive compounds from date seeds, 3.3 g of date seeds powder were mixed with 100 ml of 70 % NADES. The mixture was sonicated using a probe-type ultrasound system (model SFX, Branson, Mexico) with 80 % amplitudes for 15 min. The temperature was maintained between 35 and 40 °C throughout the treatment time by placing the sample in a ice cold-water bath. After UAE treatment, the samples were centrifuged at 10, 528 x*g* for 15 min at 4℃. The supernatant was filtered and stored at −20 °C until used for further analyses which were carried out within 2 weeks.

### 2.5 Viscosity measurement

The viscosity of prepared NADESs was analyzed using a Brookfield digital viscometer (AMETEX, USA) operated at a speed of 100 rpm with spindle number 6. The temperature was maintained by a hot water bath connected externally, each NADES sample was measured at 40 °C and 60 °C to investigate the effect of temperature change on viscosity.

### 2.6 FTIR (Fourier-transform infrared) spectroscopy

Amongst all the NADESs synthesized, the optimal NADES and its individual components were evaluated for Fourier Transform infrared spectroscopy (FTIR) using Spectrum Two FT-IR Spectrophotometer (103146, PerkinElmer, UK). The sample to be analyzed was placed over the light path, the respective interference was measured as a transform for wavenumber from 400 to 4000 cm^−1^ at a resolution of 4 cm^−1^.

### 2.7 Determination of total phenolic content (TPC) and total flavonoid content (TFC)

The various synthesized NADES were used to analyze for TPC by the method of [40] with slight modifications. The analysis was performed in triplicates and TPC was expressed as milligrams of Gallic acid equivalents per gram of powder (mg GAE/g^−^ powder). For complete details of the method, kindly refer to the supplementary material (**Section: 1**).

Total flavonoid content (TFC) was estimated with the aluminum chloride assay described by Zhishen et al. [41]. The analysis was performed in triplicates and the results were expressed in mg quercetin equivalents per gram of DSP (mg QE/g powder). For complete details of the method, kindly refer to the supplementary material (**Section: 1**).

### 2.8 Determination of antioxidant activity

The radical scavenging activity 1,1-diphenyl-2-picrylhydrazyl (DPPH) of the NADES-based date seed extracts was conducted as described by Mostafa, Airouyuwa and Maqsood [42]. The analysis was performed in triplicates and the results were expressed in mmol Trolox equivalents per gram of DSP (mmol TE/g powder). The detailed methodology is in the supplementary material **(section: 2).**

The ferric reducing antioxidant power (FRAP) assay was analyzed by the method of [21]. The analysis was performed in triplicates and the results were expressed in mmol Trolox equivalents per gram of DSP (mmol TE/g powder). The detailed methodology is in the supplementary material (**section: 2**).

The ABTS scavenging assay was analyzed following the method described by Mostafa et al. [42] with slight modifications. The analysis was performed in triplicates and the results were expressed in mmol Trolox equivalents per gram of DSP (mmol TE/g powder). The detailed methodology is in the supplementary material (**section: 2**).

### 2.9 Response surface methodology for optimization of ultrasound-assisted extraction (UAE) of phenolic compounds from date seeds powder

The UAE was designed using design expert software (version 11.0, Stat Ease Inc., Minneapolis, USA) in which response surface methodology (RSM) and Box-Behnken Design (BBD) were utilized to estimate the optimum extraction conditions required to yield the highest TPC and DPPH radical scavenging activity. The experiment comprises 27 runs with the factors; time (X_1_) (10, 20, and 30 °C), amplitude (X_2_) (70, 80, and 90 %), % NADES content (X_3_) (30, 50, and 70 %) and solid/solvent ratio (X_4_) (1:30, 1.5:30, 2:30 g/ml) were selected based on the factorial design as shown in Table 2. The experimental analyses were evaluated in triplicates and the results were fitted to the second-order polynomial equation as shown below:

(1) $$Y=\beta_{0}+\beta_{1}X_{1}+\beta_{2}X_{2}+\beta_{3}X_{3}+\beta_{4}X_{4}+\beta_{11}X_{1}+\beta_{22}X_{2}+\beta_{33}X_{3}+\beta_{44}X_{4}+\beta_{12}X_{1}X_{2}+\beta_{13}X_{1}X_{3}+\beta_{14}X_{1}X_{4}+\beta_{23}X_{2}X_{3}+\beta_{24}X_{2}X_{4}+\beta_{34}X_{3}X_{4}+\varepsilon$$

Where Y represents the forecasted responses for TPC and DPPH, β_0_ is the intercept, β_1_, β_2_, β_3_ and β_4_ are the linear regression coefficient, β_12_, β_13_, β_14,_ β_23_ and β_34_ represent the interactions while β_11_, β_22_, β_33,_ and β_44_ represent the quadratic coefficients. The generated 3D surface plots from the polynomial equation were used to interpret the correlation between the dependent variables (TPC and DPPH radical scavenging activity) and each independent UAE variables.

**Table 2:** Box-Behnken Design with experimental results for ultrasound-assisted extractions of DSP with NADES (choline chloride-lactic acids).

Independent variables	Levels		
	−1	0	1
Time (min)	10	20	30
Amplitude (m)	70	80	90
NADES Content (%)	30	50	70
Solid to Solvent Ratio (g/mL)	1:30	1.5:30	2:30

Run Order	Time (min)	Amplitude (%)	% NADES contents	Solid/solvent ratio	TPC(ChCl-LA) (mg GAE/g DSP)	DPPH (ChCl-LA (mMol TE/g DSP)
1	20	90	50	0.20	98.96±0.55^o^	505.34±1.71*^n^*
2	10	80	70	0.15	125.21±1.14^g^	612.05±1.64^j^
3	20	80	50	0.15	114.80±3.30^j^	633.11±2.26^g^
4	20	80	70	0.10	152.69±1.04^a^	747.05±0.20^a^
5	20	90	30	0.15	89.92±0.77^r^	351.73±2.08*^t^*
6	10	90	50	0.15	101.67±0.39*^n^*	545.77±3.50^l^
7	10	80	30	0.15	98.97±1.27^o^	384.44±4.26^s^
8	20	70	50	0.20	104.72±1.25*^m^*	465.81±3.20*^n^*
9	20	90	50	0.10	107.95±2.52^l^	675.39±3.94^e^
10	30	80	50	0.10	111.42±3.61^k^	711.43±2.13^c^
11	20	70	70	0.15	130.39±0.51^e^	616.67±2.84^i^
12	20	80	50	0.15	119.85±3.32^h^	632.49±4.53^g^
13	30	80	70	0.15	136.07±1.69^b^	685.52±1.11^d^
14	20	70	30	0.15	101.06±0.64*^n^*	385.52±3.04^q^
15	20	70	50	0.10	128.70±2.44^f^	737.12±2.88^b^
16	30	80	30	0.15	93.42±1.20^p^	317.23±1.84^u^
17	10	80	50	0.20	103.86±3.18*^m^*	402.77±1.03^r^
18	20	80	50	0.15	119.85±3.32^h^	632.42±2.45^g^
19	20	80	30	0.20	92.35±1.37^r^	285.86±0.26^v^
20	20	80	30	0.10	98.96±0.55^o^	505.34±1.71*^m^*
21	30	80	50	0.20	134.39±1.67^d^	457.01±2.38^o^
22	10	80	50	0.10	107.95±2.62^l^	685.39±2.82^d^
23	20	90	70	0.15	136.10±1.07^c^	633.48±2.25^f^
24	30	90	50	0.15	116.24±0.88^i^	450.85±1.17^p^
25	10	70	50	0.15	107.76±0.46^l^	405.86±1.40^q^
26	30	70	50	0.15	137.19±3.24^b^	621.20±0.58^h^
27	20	80	70	0.20	134.68±1.26^d^	548.17±2.08^k^

### 2.10 Identification and quantification of the major phenolic compounds in date seed powder

The Identification and quantification of the phenolic compounds extracted from date seed powder was done following the method described by Mostafa, Hamdi, Airouyuwa and Maqsood [1], 200 µL of the phenolic extracts using both ChCI-LA and conventional solvents were filtered through a 0.45 µm micropore membrane (PTFE, Waters, Milford, MA, USA) then were injected into UHPLC-PDA. For complete details of the methodology, kindly refer to the supplementary material (Section: 3).

### 2.11 Statistical analysis

The experiments were carried out in triplicate. All the results were presented as mean ± SD and data were analyzed using the SPSS software (version 23). The RSM data was analyzed using design expert software (trial version 13.0, Stat Ease Inc., Minneapolis, USA). Analysis of variance (ANOVA) was applied to determine the linear regression, quadratic coefficients and interactions. The coefficient of estimation of R^2^ and the adjusted coefficient of determination (adjusted R^2^) based on the polynomial equations were estimated at 95 % (*P* < 0.05) significant levels.

## 3 Results and discussion

### 3.1 Viscosity of the synthesized NADES

Viscosity is an important physical characteristic of a fluid and it estimates the internal resistance force within the fluid relative to motion. The viscosity of the seven different NADES were evaluated as a function of temperatures (40 °C and 60 °C) Table 1. The increase in temperature from 40 °C to 60 °C shows a decrease in viscosity, which indicates expansion in the molar volumes as a result of the increase in kinetic energy and the free molecular movement between the HBD and HBA [43], [44]. The state of HBD at room temperature plays a significant role in the viscosity of NADES. Similarly, the viscosity of the liquid HBD at room temperature also impacts the final viscosity of the NADESs. For instance, polyalcohol-based HBD (1,4-Butanediol) and lactic acid were fluidic at room temperature and had lower viscosity than HDBs which were solid at room temperature. While an increase in temperature from 40 °C to 60 °C did not have any significant effect on the viscosity of the polyalcohol-based NADES (choline chloride-1,4-Butanediol). Whereas, in the carboxylic acid-based NADES (choline chloride-lactic acid), there was a slight decrease in the viscosity as temperature increased from 40 °C to 60 °C with the viscosity of 20 cP and 10 cP respectively; the differences in the two HBD could be as a result of individual viscosity at room temperature. Furthermore, HBDs which were solid at room temperature were greatly influenced as temperature increased. For instance, the viscosity of NADES with a solid HBD (malic acid) at room temperature was 1710 cP at 40 °C, an increase in temperature to 60℃ the viscosity was reduced to 480 cP; which indicates expansion in the molar volumes as a result of the increase in kinetic energy and the free molecular movement between the HBD and HBA [44].

Among the sugar-based HBD, sucrose had the highest viscosity which may be due to its characteristics as a disaccharide with more hydrogen bonds compared to glucose a monosaccharide. Gómez, Tadini, Biswas, Buttrum, Kim, Boddu and Cheng [45], revealed that the high viscosity of NADES is attributed to a large number of hydrogen bonds interaction between the HBD and HBA. An increase in temperature of the NADES molecules leads to a corresponding decrease in the internal resistance force between the NADES molecules which results in low viscosity [44]. Similarly, the addition of water helps to decrease the viscosity of NADES molecules [11], [17]. One of the challenges associated with the applications of NADES on a large scale is viscosity, a high viscous solvent hinders the rate of mass transfer [11], [17], [46], nevertheless, NADES with high viscosity can be overcome by the addition of correct amount of water and increasing the temperature which can reduce the viscosity of the NADES and facilitate its transfer throughout the matrix.

### 3.2 FTIR spectrum of the synthesized NADES showing optimal extraction efficiency

**Fig. 1:**
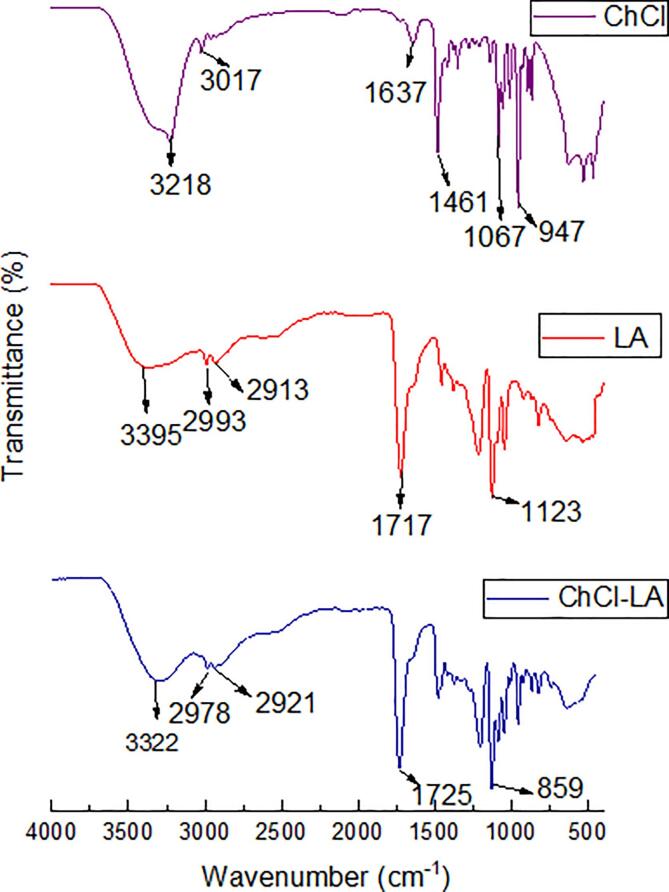
FTIR spectra of NADES and its constituents. The purple spectra represent hydrogen bond acceptor (HBA) (ChCl), red spectra represent hydrogen bond donor (HBD) (LA) and blue spectra represent NADES (ChCl-LA).

A Fourier-transform infrared spectroscopy study was carried out on the NADES with the optimal extraction efficiency (ChCl-LA) and its components to investigate the structural changes that occur between the HBD and HBA of the NADES (ChCl-LA) constituents. The FTIR spectral of ChCL (purple spectrum), LA (red spectrum), and ChCl-LA (blue spectrum) were presented in Fig. 1. The investigated ChCl spectrum revealed that the fingerprint region had a prominent peak observed at the C—N bond that shifted from 1067 to 947 cm^−1^, the C—H bending was detected at 1463 cm^−1^, the N—H peak was observed at 1637 cm^−1^ and the most prominent peak in the functional group region was the O—H stretches at 3218 cm^−1^. A similar spectrum was observed by Lanjekar and Rathod [47] in the green extraction of Glycyrrhizic acid from *Glycyrrhiza glabra* using choline chloride-based natural deep eutectic solvents (NADESs). In the LA spectrum, one of the most prominent peaks of the functional group region was the O—H stretches with a wavenumber of 3395 cm^−1^ and a minor peak representing the C=H double bond molecule was observed at 2993–2913 cm^−1^. In the study of Păucean, Vodnar, Mureșan, Fetea, Ranga, Man, Muste and Socaciu [48], lactic acid spectrum displayed prominent peaks between 1800 and 600 cm^−1^ and a peak at 1717 cm^−1^ which was due to C=O stretch representing carboxylic acid group. Similar peaks were also reported in the lactic acid spectrum obtained in the present study. The fingerprint region with wavenumber 1123 cm^−1^ belonging to carboxylic acid group is used to distinguish lactic acid from other products [49]. To compare the individual NADES components with the synthesized NADES, it was observed that two main peaks undergo structural and conformational changes as shown in the blue spectrum in Fig. 1. First, a longer stretch was observed that is the O—H peak of the ChCl-LA with a wavenumber 3322 cm^−1^, this could be due to the hydrogen bond interaction between ChCl and LA [50]. Moreover, the unique peak that differ lactic acid from other products was reduced from 1123 to 859 cm^−1^ indicating a structural change that may have resulted from the combination of the lactic acids and choline chloride.

### 3.3 Screening for the optimal NADESs for recovery of bioactive compounds from date seeds

**Fig. 2:**
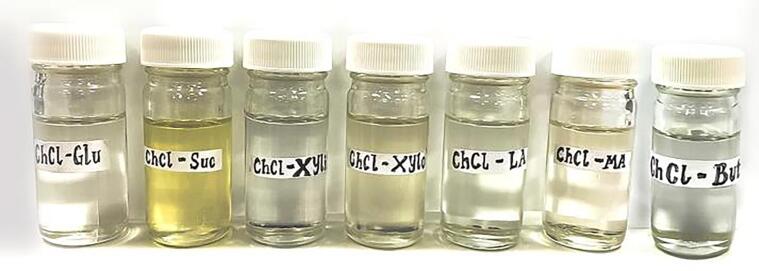
The various NADES used in this study. From the left to right: Choline chloride-Glucose (ChCl-Glu), Choline chloride -Sucrose (ChCl-Suc), Choline chloride-Xylitol (ChCl-Xyli), Choline chloride-Xylose (ChCl-Xylo), Choline chloride-Lactic acid (ChCl-LA), Choline chloride-Malic acid (ChCl-MA), choline-1,4 Butanediol (ChCl-But).

All the various NADES were presented in Fig. 2, the NADES with sugar-based hydrogen donors were very viscous and transparent (glucose, xylitol and xylitol) with the exception of sucrose which was viscous and faintly yellow. Similarly, the carboxylic acid-based and polyalcohol-based NADES were all transparent in appearance. The effect of UAE on the date seed powder for optimal recovery of total phenolic compounds (TPC) using seven different NADES was presented in Fig. 3a.The improvement in the recovery of the bioactive compounds when utilizing UAE is generally due to the acoustic cavitation which generate bubbles and cause a rise in pressure and temperature as the bubbles collapse there is a disruption in the date seed cell wall matrix permitting the release of the bioactive compounds [8] The effect of using UAE includes cell wall disruption, facilitate solvent penetration and allow the release of bioactive compounds effectively [8], [51]. Also, the ability of NADES to extract bioactive compounds is related to its physicochemical characteristics such as viscosity, pH, polarity, and the type of interactions between the hydrogen bond donor and acceptors [52]. The NADES utilized in the study are the ChCl-based NADESs comprising of carboxylic acid-based HBD, sugar-based, and polyalcohol-based HBD as presented in Table 1. Seven different NADESs were applied at 70 % concentration with the addition of 30 % water in order to reduce the viscosity, enhance the mass transfer rate, improved extraction yield, and better handling of the solvents [10], [53]. In this study, the highest recovery of total polyphenolic compounds was obtained in the carboxylic acid-based HBD, followed by sugar-based and polyalcohol-based HBD (ChCl-LA = ChCl-MA > ChCl-Xylo = ChCl-Suc > ChCl-Xyli = ChCl-Glu > ChCl-But with the yield varies from 125.26 ± 3.31 to 76.57 ± 2.96 mg GAE/g powder (Fig. 3a). The differences in the results may be due to the different structural bonds between the NADES molecules and their interaction with the phenolic compounds [54], [55]. Furthermore, carboxylic acid-based NADES are more polar compared to sugar and polyalcohol-based NADESs which resulted in a higher recovery rate of TPC [17], hence greater extraction yield was achieved by carboxylic-based HBD [11], [56]. In addition, the pH of the various NADESs also contributes to the yield of TPC as polar and more acidic NADESs result in a higher recovery rate of polyphenolic compounds [10], [43]. Similarly previous studies has reported that during the extraction of phenolic compounds from Chinese dark tea, carboxylic-based NADES had the optimal yield [57]. Furthermore, for the extraction of flavonoids from DSP, the highest yield was found in the order of (ChCl-LA = ChCl-But > ChCl-Xylo > ChCl-MA = ChCl-Xyli > ChCl-Suc = ChCl-Glu (Fig. 3a). It was previously reported that highest extraction or recovery of the total flavonoids from sour cherry pomace was found to be in carboxylic acid-based NADES [58]. In addition, NADES with branched-chain polyalcohol is also suitable for the extraction of less polar compounds like flavonoids [59], [60], which could be the reason for the high recovery with ChCl-But in this study. The total flavonoid content (TFC) from date seed powder showed no significant difference (P > 0.05) among the carboxylic acid (ChCl-LA) and polyalcohol (ChCl-But)-based HBD with corresponding values of 79.99 ± 1.08 and 80.01 ± 0.85 mg QE/g powder, respectively. Similar results were found for the extraction of flavonoids using lactic acid and 1,2-propanediol as HBD from inflorescences of *Helichrysum arenarium* L. [60].

**Fig. 3a:**
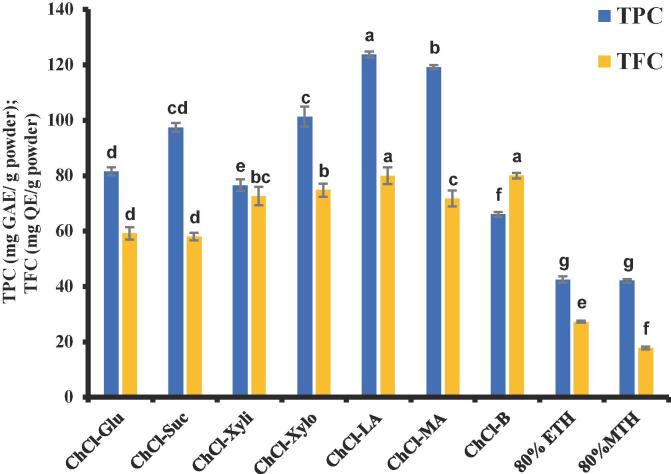
Total polyphenol content (TPC) (mg GAE/g powder) and total flavonoid content (TFC) (mg QE/g powder) of date seeds extracted with 70 % NADES 80 % ethanol and methanol. Different letters on the bars showed significant differences among different samples (p < 0.05). Value represents mean ± SD (n = 3).

The antioxidant activity of date seed powder extracted with green solvents was studied in relation to bioactive compounds since they are significantly involved in the antioxidant activity of natural products [10], [61], [62] This study investigated the antioxidant activity of the different NADESs extracts using three antioxidant assays (ABTS radical scavenging activity, DPPH radical scavenging activity, and FRAP activity). It is pertinent to note that amongst the different HBD utilized, the carboxylic-based HBD (ChCl-LA) showed higher antioxidant activity compared to the other extraction solvents as shown in Fig. 3b.

**Fig. 3b:**
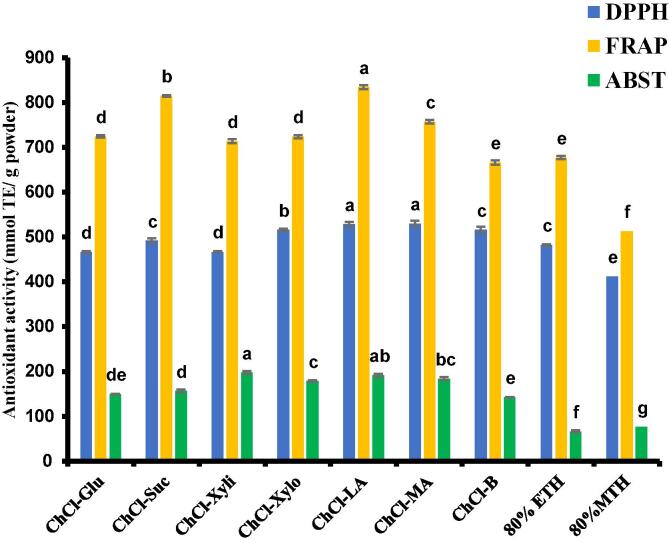
Antioxidant activity (ABTS, DPPH radical scavenging activity and FRAP) (mmol TE/g powder) of date seeds extracted with 70 % NADES 80 % ethanol and methanol. Different letters on the bars showed significant differences among different samples (p < 0.05). The Value represents mean ± SD (n = 3).

Also, the extraction efficiency of NADESs was compared with conventional solvents. The extraction was conducted with 80 % ethanol and 80 % methanol as reference solvents using the same extraction techniques with NADESs. The result agrees with previous studies which suggest that NADESs could be used as an efficient substitute for conventional solvents [10], [25], [57]. The NADESs have the ability to form a hydrogen bond between the target molecules to be extracted, by donating and accepting electrons consequently increasing the rate of mass transfer [10], [54]. In this study, all the NADESs had significantly higher extraction potentials (P < 0.05) compared to the conventional solvents (ethanol and methanol). From the results of bioactive compounds extraction and antioxidant activity of date seeds using 7 different NADES and UAE, it was observed that carboxylic acid-based HBD (ChCl-LA) had the highest extraction yield and was further utilized in the optimization process.

### 3.4 Model fitting

**Fig. 4:**
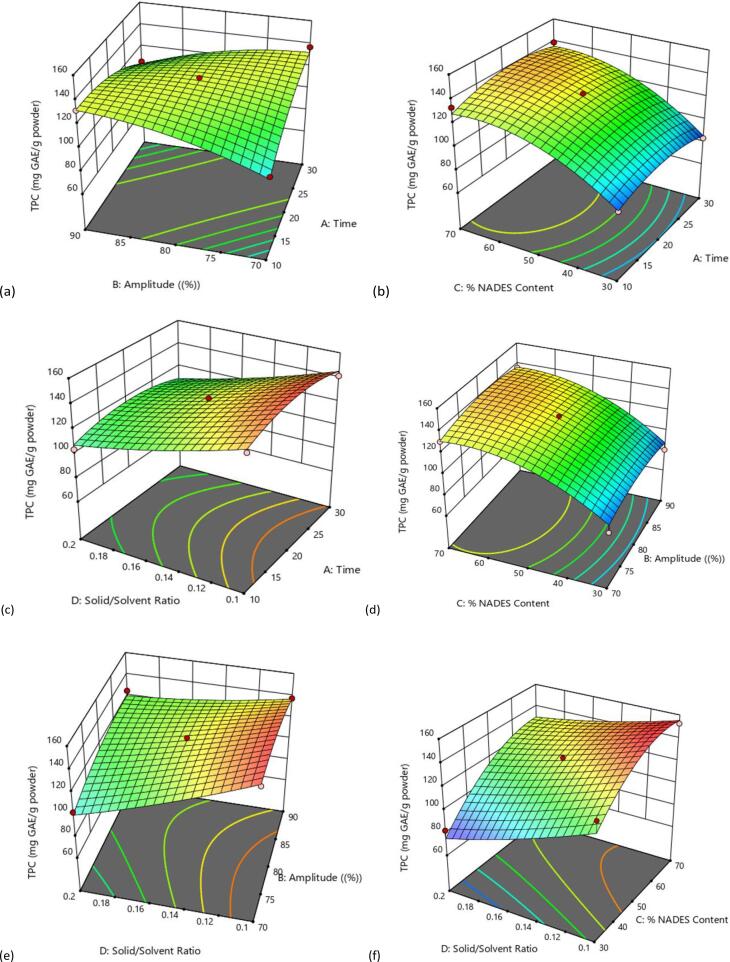
Response surface plot showing the combined effect of ultrasound power and time (a), percentage NADES contents and time (b), solid/solvent ratio and time (c) percentage NADES content and ultrasound power (d), solid/solvent ratio and ultrasound power (e) and solid/solvent ratio and percentage NADES contents (f) on the total phenolic content (TPC) of date seeds extracted with NADES (ChCl-ssLA).

The results from the experimental modeling indicated in Table 2, that the TPC and DPPH scavenging activity ranged from 89.92 ± 0.77–152.69 ± 1.04 mg GEA/g DSP and 285 ± 0.26 ± 747.05 ± 0.20 mmol TE/g DSP, respectively. The results from the ANOVA table revealed that the model was highly significant (P < 0.0001), while the lack of fit of each model was not significant (P > 0.05) revealing that the model adequately described the relationship between the independent variables (extraction time, solid to solvent ratio, % NADES content and amplitudes) and the dependent variables (TPC and DPPH). The R^2^ and the adjusted R^2^ of all the responses were >0.92, indicating a good model with a high degree of correlation between the experimental and predicted values Table 3**.** The three-dimensional (3D) response surface plots corresponding to the interactions among the variables were presented in Fig. 4.

**Table 3:** Regression coefficient values generated for the antioxidant capacity of the date seed phenolic extract.

Source	TPC					DPPH radical scavenging activity				
	SS	DF	MS	F-value	P-value	SS	DF	MS	F-value	P-value
**Model**	14524.18	14	1037.44	23.91	<0.0001^a^	5.794E + 05	14	41382.32	31.80	<0.0001^a^
X_1_-Time	31.07	1	31.07	0.72	0.4140	3569.37	1	3569.37	2.74	0.1236
X_2_- Amplitude	21.39	1	21.39	0.49	0.4960	391.48	1	391.48	0.3008	0.5934
X_3_-% NADES Content	7176.56	1	7176.56	165.39	<0.0001^a^	2.742E + 05	1	2.742E + 05	210.74	<0.0001^a^
X_4_-Solid/Solvent	4283.12	1	4283.12	98.71	<0.0001^a^	2.125E + 05	1	2.125E + 05	163.31	<0.0001^a^
X_1_X_2_	987.84	1	987.84	22.77	0.0005^b^	24065.32	1	24065.32	18.49	0.0010^b^
X_1_X_3_	17.68	1	17.68	0.41	0.5352	4947.72	1	4947.72	3.80	0.0749
X_1_X_4_	2.16	1	2.16	0.05	0.8272	198.81	1	198.81	0.1528	0.7027
X_2_X_3_	8.56	1	8.56	0.19	0.6649	613.06	1	613.06	0.4711	0.5055
X_2_X_4_	306.08	1	306.08	7.05	0.0209^c^	2563.40	1	2563.40	1.97	0.1858
X_3_X_4_	0.4900	1	0.4900	0.01	0.9171	8028.16	1	8028.16	6.17	0.0288^c^
X_1_2	461.20	1	461.20	10.63	0.0068^c^	17366.53	1	17366.53	13.35	0.0033^c^
X_2_2	288.94	1	288.94	6.66	0.0241	9767.91	1	9767.91	7.51	0.0179^c^
X_3_2	828.73	1	828.73	19.10	0.0009^b^	17960.41	1	17960.41	13.80	0.0030^c^
X_4_2	96.83	1	96.83	2.23	0.1611	3022.71	1	3022.71	2.32	0.1534
Residual	520.70	12	43.39			15615.95	12	1301.33		
Lack of Fit	511.86	10	51.19	11.59	0.0820	11415.16	10	1141.52	0.5435	0.7913
R^2^	0.9654					0.9738				
Adjusted R^2^	0.9250					0.9431				
Predicted R^2^	0.8039					0.8736				

X_1_-Extraction time (minutes), X_2_- Ultrasound amplitude, X_3_-% NADES content, X_4_-Solid/solvent ratio, SS-Sum of squares, DF-Degrees of freedom, MS-Mean square, R_2_-Quadratic correlation coefficient. ^a^- (p < 0.0001), ^b^-(p < 0.001), ^c^-(p < 0.05).

### 3.5 Effect of the independent variables on the dependent variable (TPC)

The effect of independent variables on TPC as presented in Table 3. The effect of % NADES content (X_3_) and the solid-to-solvent ratio (X_4_) in the extraction of phenolic compounds was significant with contributions of 70 % and 1:30 g/ml, respectively. The % NADES content had a positive significant effect (P < 0.001), which reveals that higher % NADES content was able to extract more bioactive compounds from the date seeds. Also, the different levels of % NADES content have different extraction efficiency indicating the role of water in the extraction of phenolic compounds. The highest extraction efficiency was observed with 70 % NADES content, the solid to solvent ratio of 0.03 g/ml at 80 % US amplitudes and the extraction time of 20 mins as shown in Table. 2**.** These results indicates that the water content present in the NADES changes the polarity and viscosity of NADES leading to differences in the extraction yield. Studies have shown that the amount of water in NADES determines the extraction efficiency of bioactive compounds [56], [63], [64], [65], [66]. In the ultrasound-assisted extraction of phenolic compounds from *Lavandula angustifolia* flowers using NADES (choline chloride: glycerol) at a molar ratio of 1:2, the optimal % NADES content was 66.5 %, the amplitudes, liquid to solvent ratio, and extraction time was 60 %, 31.7 ml/g, and 17.5 min respectively [67]. Similarly, in the extraction of phenolic compounds from *Moringa oleifera* leaves, the optimal %NADES was 63 % when extraction was carried out at temperature and ultrasonic power of 40 °C and 144 W, respectively [56]. Similarly, it is important to consider the volume of NADES and the mass of the natural product to be extracted, as large quantity of solvents do not support the extraction principle of green chemistry. A large volume of solvent could result in wastage and complication of experimental design, however, low quantity of solvents may lead to partial extraction of the compounds of interest. The rate of mass transfer of bioactive compounds is influenced by the solid-to-solvent ratio [68]. The quadratic effects of X_1_^2^, X_2_^2^, and X_3_^2^ showed a significant effect on TPC. In addition, the significant interactive effects of the independent variables on TPC were observed between the extraction time (X_1_) and the amplitude (X_2_), and between amplitude (X_2_) and solid-to-solvent ratio (X_4_). Equation (2) represents the effect of the independent variables on TPC.

(2) $$Y_{\mathrm{TPC}\;}=131.5+1.61X_{1}+1.34X_{2}+22.79X_{3}-17.23X_{4}-15.72X_{1}X_{2}+2.10X_{1}X_{3}-0.74X_{1}X_{4}+1.46X_{2}X_{3}+8.75X_{2}X_{4}+4.65X_{3}X_{4}-\;8.47X_{1}^{2}-6.53X_{2}^{2}-14.13X_{3}^{2}+2.59X_{4}^{2}\;$$

To describe the interaction independent variables had with TPC, the 3D surface plots were developed as presented in Fig. 4. The ultrasound amplitude and extraction time were increased at a fixed % NADES content and solid to solvent ratio Fig. 4a**.** The recovery of TPC from date seeds significantly increased at the upper limit of amplitude and slightly above the lower limit of the extraction time, which support the fact that higher extraction time may oxidize phenolic compounds resulting in degradation of phenolic compounds [64], [69]. The UAE increases the extraction yield of bioactive compounds by disrupting the intracellular membrane and facilitating solvent penetration into the plant matrix [64], [70]. Nevertheless, higher extraction time may oxidize phenolic compounds resulting in degradation of TPC [64], [69]. In the present study, Fig. 4e shows the interactions between the solid-to-solvent ratio and amplitude at a fixed extraction time and % NADES content. The results showed that extracted phenolics increased as the US amplitude increased, demonstrating the optimal value at 90 %. However, the increase in solid-to-solvent ratio decreases the TPC from date seed powder, and the optimal extraction from date seed powder was observed at the lowest solid-to-solvent ratio (0.03 g/ml). The reason for the maximum extraction yields of phenolic compounds at 0.03 g/ml could be as a result of the NADESs molecules having a maximum molecular interaction between the phenolic compounds, penetrating all the cell walls of the date seed powder. Thus, when the solid to liquids ratio were increased the molecular interactions between the NADES and the phenolic compound might have weakened resulting in a decrease in the extraction efficiency. The rate of diffusion of the solvent into the intracellular membrane is determined by the solid-to-solvent ratio [68]. In general, a lower solid-to-solvent ratio has more potential to penetrate and dissolve the biomolecules resulting in a higher extraction yield. In addition, the hydrogen bond interactions within the NADES lead to an increase in the extraction yield of the bioactive compounds [71], [72]. In the extraction of flavonoids from *Oroxylum indicum* seeds using chloride:1,4-butanediol-based DES, the liquid to solid ratio experience a gradual increase from 5:1 to 20:1 with optimal extraction yield in the four flavonoids (oroxin B, oroxin A, balcalein, and chrysin) being achieved at 20:1. However, increasing the liquid to solid ratio from 40:1 to 100:1 the extraction yields were reduced [68]. The consideration of solid to solvent ratio as a factor when optimizing the extraction of bioactive compounds using DES/NADES cannot be over emphasized as it is one of the major factors influencing the extraction efficiency.

### 3.6 Effect of the independent variables on DPPH radical scavenging activity

**Fig. 5:**
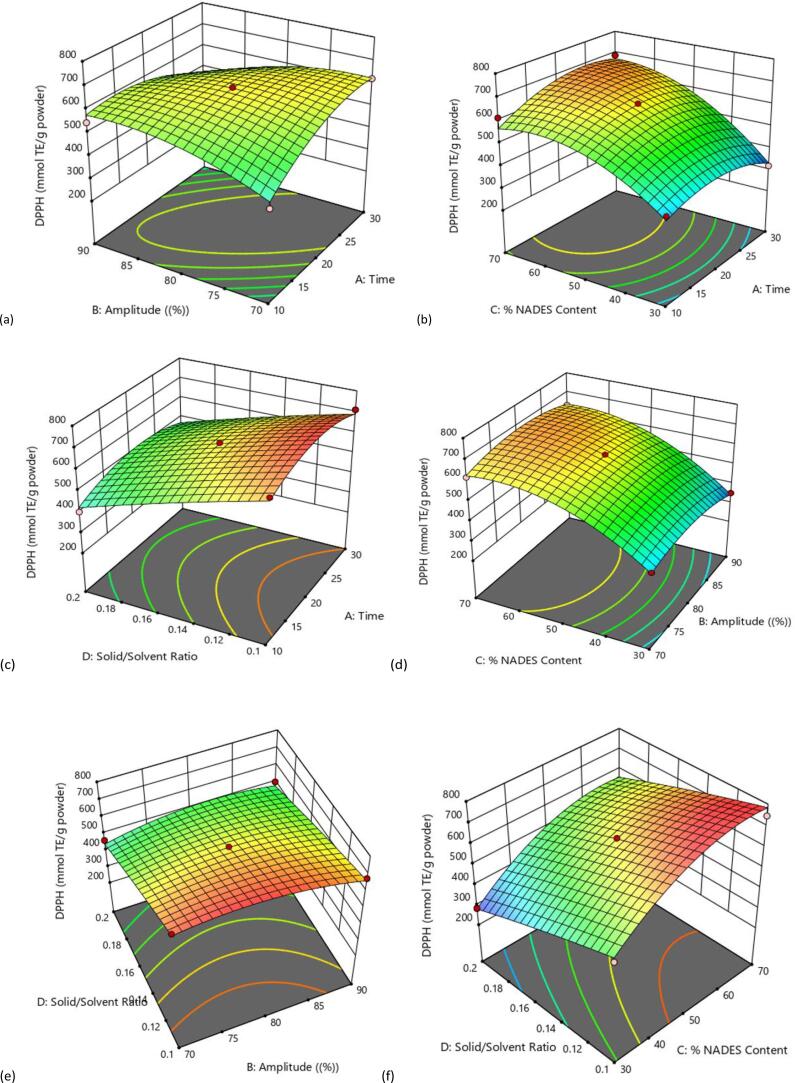
Response surface plot showing the combined effect of ultrasound power and time (a), percentage NADES contents and time (b), solid/solvent ratio and time (c) percentage NADES content and ultrasound power (d), solid/solvent ratio and ultrasound power (e) and solid/solvent ratio and percentage NADES contents (f) on DPPH radical scavenging activity demonstrated by date seeds extracted with NADES (ChCl-LA).

In this study, the effect of the independent variables on DPPH radical scavenging activity of NADES-based date seed extract was presented in the Table 3. The model was highly significant with P < 0.0001, and the DPPH radical scavenging activity was significantly influenced by % NADES content (X_3_) and solid-to-solvent ratio (X_4_) (P < 0.0001). The quadratic effects X_1_^2^, X_2_^2^, and X_3_^2^ showed a significant negative effect on DPPH radical scavenging activity. In addition, among the interactive effects of the variables on DPPH radical scavenging activity, it was observed that there were negative significant interactions between the extraction time (X_1_) and amplitude (X_2_) and also, positive significant interactions between % NADES content (X_3_) and solid-to-solvent ratio (X_4_). The DPPH antioxidant scavenging activity of this model was presented by equation (3) and the 3D surface plots were presented in Fig. 5 (a-f). In this study, a similar trend was observed in the relationship between the independent variables on TPC and DPPH radical scavenging activity with similar significant impact on the interactive effects of the 3D surface plots. This model shows a good linear relationship between the TPC and the DPPH radical scavenging activity. Some studies have shown that there is a good linear correlation between polyphenolic content and antioxidant activity [56], [63], [73].

(3) $$Y_{\mathrm{DPPH}\;}=634.36+17.25X_{1}-5.71X_{2}+134.51X_{3}-116.41X_{4}-77.57X_{1}X_{2}+35.17X_{1}X_{3}+7.05X_{1}X_{4}+12.38X_{2}X_{3}+25.32X_{2}X_{4}+5.2X_{3}X_{4}-\;62.90X_{1}^{2}-48.63X_{2}^{2}-88.86X_{3}^{2}-7.03X_{4}^{2}\;\;\;\;$$

### 3.7 Verification of the model using the optimal predicted NADES and UAE conditions

To determine the reproducibility and the reliability of the RSM design, the predicted conditions obtained from the model were experimentally verified. The conditions for the optimization of the model were obtained based on regression analysis of the variables and the 3D surface plots. The optimal conditions under which higher TPC and DPPH radical scavenging activity were achieved are extraction time (15 min), amplitudes (90 %), % NADES content (70 %), and solid-to-solvent ratio (0.03 g/ml). The predicted values for TPC and DPPH radical scavenging activity were 146.22 mg GAE/g powder and 718.57 mmol TE/g powder and the experimental values were 145.54 ± 1.54 mg GAE/g powder and 71919 ± 2.09 mmol TE/g powder. To predict the best conditions to obtain the maximum extraction efficiency for TPC and DPPH radical scavenging activity, the function scale of desirability range from 0 to 1 (0 = extremely undesirable, and 1 = extremely desirable response). In this study, the desirability function of the optimized model was 0.97. The experimental results reported were close to the predicted results generated using the RSM which validated the model.

### 3.8 HPLC analysis of the phenolic compounds present in the NADES extracted DSP

The phenolic compounds present in the date seed extracts obtained using the optimal NADES (ChCL-LA) and two conventional extraction solvents (80 % ethanol and 80 % methanol) were identified and quantified as presented in Table 4. A total of 14 different phenolic compounds were identified and quantified, 8 compounds were identified in samples extracted with NADES (ChCL-LA), 5 compounds in samples extracted with 80 % ethanol, and 6 compounds in samples extracted with 80 % methanol. The phenolic compounds identified were categorized into hydroxybenzoic (gallic acid, 3,4-Dihydroxy benzoic acid, 4 hydroxybenzoic acid, vanillic acid, syringic acid, and benzoic acid), hydroxycinnamic (ferulic acid, p-coumaric, caffeic, cinnamic acid, and chlorogenic acid) and flavanol (quercetin, catechin, and rutin). The presence of these phenolic compounds corresponds with the previous HPLC-based analysis and identification of date seed phenolic compounds [74], [75]. The primary phenolic compounds from ChCL-LA extract were 3,4-Dihydroxy benzoic, catechin and caffeic. In 80 % ethanol extract, the major phenolic compound was benzoic acid, and 3,4-Dihydroxy benzoic, whereas, in 80 % methanol extract, gallic acid followed by catechin were the major phenolic compounds. The 3,4-Dihydroxy benzoic acid which was one of the highest quantified phenolic compounds is considered to possess good antioxidant activities [42], [76].

**Table 4:** Phenolic compounds identified in DSP extract extracted with Choline chloride: lactic acid (ChCl-LA), 80% ethanol, and 80% Methanol coupled with UAE.

Phenolic compounds (mg/100 g DSP)	Sample extraction conditions		
	Choline chloride: lactic acid (ChCl-LA)	80 % Ethanol	80 % Methanol
Gallic acid	N/D	N/D	380.04 ± 0.21
3,4-Dihydroxybenzoic acid	455.87 ± 0.19	96.41 ± 0.03	100.31 ± 0.11
Catechin	152.04 ± 0.08	N/D	124.86 ± 0.0.26
4-hydroxybenzoic acid	16.25 ± 0.01	N/D	78.83 ± 0.01
Vanillic acid	15.38 ± 0.09	34.59 ± 0.08	18.42 ± 0.02
Caffeic acid	152.22 ± 0.08	N/D	N/D N/D
Syringic acid	15.40 ± 0.12	39.88 ± 0.1	N/D
p-coumaric acid	N/D	N/D	N/D
Ferulic acid	N/D	N/D	N/D
Rutin hydrate	15.25 ± 0.14	N/D	N/D
Cinnamic acid	N/D	N/D	N/D
Quercetin	15.33 ± 0.04	N/D	N/D
Benzoic acid	N/D	206.95 ± 0.21	N/D
Chlorogenic acid	N/D	38.47 ± 0.13	61.08 ± 0.01

## 4 Conclusion

This study reveals that NADES coupled with UAE present a sustainable and efficient green extraction approach for the optimal recovery of bioactive compounds with high antioxidant activity from date seed powder compared to conventional extraction solvents. Out of the seven NADES investigated in this study, ChCl-LA had the highest extraction efficiency for the recovery of the bioactive compounds and demonstrated highest antioxidant activity. The application of Box-Behnken design in the optimization process showed that % NADES content (70 %) and solid-to-solvent ratio of 1:30 g/mL were the main factors influencing the optimal extraction yield of phenolic compounds and DPPH radical scavenging activity. The optimal experimental conditions for attaining the maximum recovery of phenolic as reflected by TPC (145.54 ± 1.54 mg GAE/g powder) and DPPH radical scavenging activity (719.19 ± 2.09 mmol TE/g powder) were extraction time of 15 min, US amplitude of 90 %, % NADES content of 70 % and solid-to-solvent ratio of 1:30 g/ml. While the predicted values for TPC and DPPH radical scavenging activity were 146.22 (mg GAE/g powder) and 718.57 (mmol TE/g powder), respectively. Catechin, 3,4-dihydroxybenzoic acid, and caffeic acid were the major polyphenolic compounds present in the date seed extracts. This study also illustrates the importance of date seed valorization and the use of sustainable and eco-friendly green extraction techniques for recovery of bioactive compounds with high antioxidant activity which could find useful applications in the food, pharmaceutical, and cosmetics industries.

## Declaration of Competing Interest

The authors declare that they have no known competing financial interests or personal relationships that could have appeared to influence the work reported in this paper.

## References

[1] Mostafa H., Hamdi M., Airouyuwa J.O., Maqsood S. (2022). Efficient valorization of date fruit processing by-product through nano-and green-extraction technology: a response surface methodology-based optimization study. Biomass Convers. Biorefin..

[2] Echegaray N., Pateiro M., Gullon B., Amarowicz R., Misihairabgwi J.M., Lorenzo J.M. (2020). Phoenix dactylifera products in human health–A review. Trends Food Sci. Technol..

[3] Bouaziz F., Ben Abdeddayem A., Koubaa M., Ellouz Ghorbel R., Ellouz Chaabouni S. (2020). Date seeds as a natural source of dietary fibers to improve texture and sensory properties of wheat bread. Foods.

[4] Al-Farsi M., Alasalvar C., Al-Abid M., Al-Shoaily K., Al-Amry M., Al-Rawahy F. (2007). Compositional and functional characteristics of dates, syrups, and their by-products. Food Chem..

[5] Baliga M.S., Baliga B.R.V., Kandathil S.M., Bhat H.P., Vayalil P.K. (2011). A review of the chemistry and pharmacology of the date fruits (Phoenix dactylifera L.). Food Res. Int..

[6] Ranasinghe M., Manikas I., Maqsood S., Stathopoulos C. (2022). Date components as promising plant-based materials to be incorporated into baked goods—A review. Sustainability.

[7] Habib H.M., Platat C., Meudec E., Cheynier V., Ibrahim W.H. (2014). Polyphenolic compounds in date fruit seed (Phoenix dactylifera): characterisation and quantification by using UPLC-DAD-ESI-MS. J. Sci. Food Agric..

[8] Chanioti S., Tzia C. (2018). Extraction of phenolic compounds from olive pomace by using natural deep eutectic solvents and innovative extraction techniques. Innov. Food Sci. Emerg. Technol..

[9] González C.G., Mustafa N.R., Wilson E.G., Verpoorte R., Choi Y.H. (2018). Application of natural deep eutectic solvents for the “green” extraction of vanillin from vanilla pods. Flavour Fragr. J..

[10] Radošević K., Bubalo M.C., Srček V.G., Grgas D., Dragičević T.L., Redovniković I.R. (2015). Evaluation of toxicity and biodegradability of choline chloride based deep eutectic solvents. Ecotoxicol. Environ. Saf..

[11] Wei Z., Qi X., Li T., Luo M., Wang W., Zu Y., Fu Y. (2015). Application of natural deep eutectic solvents for extraction and determination of phenolics in Cajanus cajan leaves by ultra performance liquid chromatography. Sep. Purif. Technol..

[12] Meksi N., Moussa A. (2017). A review of progress in the ecological application of ionic liquids in textile processes. J. Clean. Prod..

[13] Smith E.L., Abbott A.P., Ryder K.S. (2014). Deep eutectic solvents (DESs) and their applications. Chem. Rev..

[14] Tang B., Zhang H., Row K.H. (2015). Application of deep eutectic solvents in the extraction and separation of target compounds from various samples. J. Sep. Sci..

[15] Zhao B.-Y., Xu P., Yang F.-X., Wu H., Zong M.-H., Lou W.-Y. (2015). Biocompatible deep eutectic solvents based on choline chloride: characterization and application to the extraction of rutin from *Sophora japonica*. ACS Sustain. Chem. Eng..

[16] Dai Y., Rozema E., Verpoorte R., Choi Y.H. (2016). Application of natural deep eutectic solvents to the extraction of anthocyanins from *Catharanthus roseus* with high extractability and stability replacing conventional organic solvents. J. Chromatogr. A.

[17] Bosiljkov T., Dujmić F., Bubalo M.C., Hribar J., Vidrih R., Brnčić M., Zlatic E., Redovniković I.R., Jokić S. (2017). Natural deep eutectic solvents and ultrasound-assisted extraction: green approaches for extraction of wine lees anthocyanins. Food Bioprod. Process..

[18] Chen J., Liu M., Wang Q., Du H., Zhang L. (2016). Deep eutectic solvent-based microwave-assisted method for extraction of hydrophilic and hydrophobic components from radix salviae miltiorrhizae. Molecules.

[19] de Oliveira A., da Conceição E., Leles M. (2016). Multiresponse optimization of an extraction procedure of carnosol and rosmarinic and carnosic acids from rosemary. Food Chem..

[20] Mansur A.R., Song N.-E., Jang H.W., Lim T.-G., Yoo M., Nam T.G. (2019). Optimizing the ultrasound-assisted deep eutectic solvent extraction of flavonoids in common buckwheat sprouts. Food Chem..

[21] Gullón B., Lú-Chau T.A., Moreira M.T., Lema J.M., Eibes G. (2017). Rutin: a review on extraction, identification and purification methods, biological activities and approaches to enhance its bioavailability. Trends Food Sci. Technol..

[22] Kehili M., Isci A., Thieme N., Kaltschmitt M., Zetzl C., Smirnova I. (2022). Microwave-assisted deep eutectic solvent extraction of phenolics from defatted date seeds and its effect on solubilization of carbohydrates. Biomass Convers. Biorefin..

[23] Dabetić N., Todorović V., Panić M., Radojčić Redovniković I., Šobajić S. (2020). Impact of deep eutectic solvents on extraction of polyphenols from grape seeds and skin. Appl. Sci..

[24] Đorđević B.S., Todorović Z.B., Troter D.Z., Stanojević L.P., Stojanović G.S., Đalović I.G., Mitrović P.M., Veljković V.B. (2021). Extraction of phenolic compounds from black mustard (*Brassica nigra* L.) seed by deep eutectic solvents. J. Food Meas. Charact..

[25] Nam M.W., Zhao J., Lee M.S., Jeong J.H., Lee J. (2015). Enhanced extraction of bioactive natural products using tailor-made deep eutectic solvents: application to flavonoid extraction from *Flos sophorae*. Green Chem..

[26] Bi W., Tian M., Row K.H. (2013). Evaluation of alcohol-based deep eutectic solvent in extraction and determination of flavonoids with response surface methodology optimization. J. Chromatogr. A.

[27] Dai Y., Witkamp G.-J., Verpoorte R., Choi Y.H. (2015). Tailoring properties of natural deep eutectic solvents with water to facilitate their applications. Food Chem..

[28] Duan M.-H., Xu W.-J., Yao X.-H., Zhang D.-Y., Zhang Y.-H., Fu Y.-J., Zu Y.-G. (2015). Homogenate-assisted negative pressure cavitation extraction of active compounds from *Pyrola incarnata* Fisch. and the extraction kinetics study. Innov. Food Sci. Emerg. Technol..

[29] Bai C., Wei Q., Ren X. (2017). Selective extraction of collagen peptides with high purity from cod skins by deep eutectic solvents. ACS Sustain. Chem. Eng..

[30] Hernández-Corroto E., Plaza M., Marina M.L., García M.C. (2020). Sustainable extraction of proteins and bioactive substances from pomegranate peel (*Punica granatum* L.) using pressurized liquids and deep eutectic solvents. Innov. Food Sci. Emerg. Technol..

[31] López R., D'Amato R., Trabalza-Marinucci M., Regni L., Proietti P., Maratta A., Cerutti S., Pacheco P. (2020). Green and simple extraction of free seleno-amino acids from powdered and lyophilized milk samples with natural deep eutectic solvents. Food Chem..

[32] Pang J., Sha X., Chao Y., Chen G., Han C., Zhu W., Li H., Zhang Q. (2017). Green aqueous biphasic systems containing deep eutectic solvents and sodium salts for the extraction of protein. RSC Adv..

[33] Gao C., Cai C., Liu J., Wang Y., Chen Y., Wang L., Tan Z. (2020). Extraction and preliminary purification of polysaccharides from *Camellia oleifera* Abel. seed cake using a thermoseparating aqueous two-phase system based on EOPO copolymer and deep eutectic solvents. Food Chem..

[34] Benvenutti L., Zielinski A.A.F., Ferreira S.R.S. (2019). Which is the best food emerging solvent: IL, DES or NADES?. Trends Food Sci. Technol..

[35] Chemat F., Abert Vian M., Ravi H.K., Khadhraoui B., Hilali S., Perino S., Fabiano Tixier A.-S. (2019). Review of alternative solvents for green extraction of food and natural products: panorama, principles, applications and prospects. Molecules.

[36] Dos Santos J.M., de Andrade J.K., Galvão F., Felsner M.L. (2019). Optimization and validation of ultrasound-assisted extraction for the determination of micro and macro minerals in non-centrifugal sugar by F AAS. Food Chem..

[37] Hammi K.M., Jdey A., Abdelly C., Majdoub H., Ksouri R. (2015). Optimization of ultrasound-assisted extraction of antioxidant compounds from Tunisian Zizyphus lotus fruits using response surface methodology. Food Chem..

[38] Rodriguez-Juan E., Rodriguez-Romero C., Fernandez-Bolanos J., Florido M.C., Garcia-Borrego A. (2021). Phenolic compounds from virgin olive oil obtained by natural deep eutectic solvent (NADES): effect of the extraction and recovery conditions. J. Food Sci. Technol..

[39] Shang X., Ma S., Pan Q., Li J., Sun Y., Ji K., Sun L. (2019). Process analysis of extractive distillation for the separation of ethanol–water using deep eutectic solvent as entrainer. Chem. Eng. Res. Des..

[40] Olatunde O.O., Benjakul S., Vongkamjan K. (2018). Antioxidant and antibacterial properties of guava leaf extracts as affected by solvents used for prior dechlorophyllization. J. Food Biochem..

[41] Zhishen J., Mengcheng T., Jianming W. (1999). The determination of flavonoid contents in mulberry and their scavenging effects on superoxide radicals. Food Chem..

[42] Mostafa H., Airouyuwa J.O., Maqsood S. (2022). A novel strategy for producing nano-particles from date seeds and enhancing their phenolic content and antioxidant properties using ultrasound-assisted extraction: a multivariate based optimization study. Ultrason. Sonochem..

[43] Mitar A., Panić M., Prlić Kardum J., Halambek J., Sander A., Zagajski Kučan K., Radojčić Redovniković I., Radošević K. (2019). Physicochemical properties, cytotoxicity, and antioxidative activity of natural deep eutectic solvents containing organic acid. Chem. Biochem. Eng. Q..

[44] Rathnasamy S.K., sri Rajendran D., Balaraman H.B., Viswanathan G. (2019). Functional deep eutectic solvent-based chaotic extraction of phycobiliprotein using microwave-assisted liquid-liquid micro-extraction from Spirulina (*Arthrospira platensis*) and its biological activity determination. Algal Res..

[45] Gómez A.V., Tadini C.C., Biswas A., Buttrum M., Kim S., Boddu V.M., Cheng H.N. (2019). Microwave-assisted extraction of soluble sugars from banana puree with natural deep eutectic solvents (NADES). Lwt.

[46] Peng X., Duan M.-H., Yao X.-H., Zhang Y.-H., Zhao C.-J., Zu Y.-G., Fu Y.-J. (2016). Green extraction of five target phenolic acids from *Lonicerae japonicae* Flos with deep eutectic solvent. Sep. Purif. Technol..

[47] Lanjekar K.J., Rathod V.K. (2021). Green extraction of Glycyrrhizic acid from *Glycyrrhiza glabra* using choline chloride based natural deep eutectic solvents (NADESs). Process Biochem..

[48] Păucean A., Vodnar D., Mureșan V., Fetea F., Ranga F., Man S., Muste S., Socaciu C. (2017). Monitoring lactic acid concentrations by infrared spectroscopy: a new developed method for Lactobacillus fermenting media with potential food applications. Acta Aliment..

[49] Huang H., Grün I., Ellersieck M., Clarke A. (2018). Use of HPLC and FTIR as a tool for analysis of lactic acid in restructured fish products. J. Nutr. Food Res. Technol.

[50] Macchioni V., Carbone K., Cataldo A., Fraschini R., Bellucci S. (2021). Lactic acid-based deep natural eutectic solvents for the extraction of bioactive metabolites of *Humulus lupulus* L.: supramolecular organization, phytochemical profiling and biological activity. Sep. Purif. Technol..

[51] Chen Y., Yin L.Z., Zhao L., Shu G., Yuan Z.X., Fu H.L., Lv C., Lin J.C. (2017). Optimization of the ultrasound-assisted extraction of antioxidant phloridzin from *Lithocarpus polystachyus* Rehd. using response surface methodology. J. Sep. Sci..

[52] Tang Y., He X., Sun J., Liu G., Li C., Li L., Sheng J., Zhou Z., Xin M., Ling D. (2021). Comprehensive evaluation on tailor-made deep eutectic solvents (DESs) in extracting tea saponins from seed pomace of Camellia oleifera Abel. Food Chem..

[53] Ruesgas-Ramón M., Figueroa-Espinoza M.C., Durand E. (2017). Application of deep eutectic solvents (DES) for phenolic compounds extraction: overview, challenges, and opportunities. J. Agric. Food Chem..

[54] Dai Y., van Spronsen J., Witkamp G.-J., Verpoorte R., Choi Y.H. (2013). Natural deep eutectic solvents as new potential media for green technology. Anal. Chim. Acta.

[55] Dai Y., Verpoorte R., Choi Y.H. (2014). Natural deep eutectic solvents providing enhanced stability of natural colorants from safflower (*Carthamus tinctorius*). Food Chem..

[56] Wu L., Li L., Chen S., Wang L., Lin X. (2020). Deep eutectic solvent-based ultrasonic-assisted extraction of phenolic compounds from *Moringa oleifera* L. leaves: optimization, comparison and antioxidant activity. Sep. Purif. Technol..

[57] Cai C., Yu W., Wang C., Liu L., Li F., Tan Z. (2019). Green extraction of cannabidiol from industrial hemp (*Cannabis sativa* L.) using deep eutectic solvents coupled with further enrichment and recovery by macroporous resin. J. Mol. Liq..

[58] Popovic B.M., Micic N., Potkonjak A., Blagojevic B., Pavlovic K., Milanov D., Juric T. (2022). Novel extraction of polyphenols from sour cherry pomace using natural deep eutectic solvents–Ultrafast microwave-assisted NADES preparation and extraction. Food Chem..

[59] García A., Rodríguez-Juan E., Rodríguez-Gutiérrez G., Rios J.J., Fernández-Bolaños J. (2016). Extraction of phenolic compounds from virgin olive oil by deep eutectic solvents (DESs). Food Chem..

[60] Ivanović M., Islamčević Razboršek M., Kolar M. (2020). Innovative extraction techniques using deep eutectic solvents and analytical methods for the isolation and characterization of natural bioactive compounds from plant material. Plants.

[61] Jokić S., Cvjetko M., Božić Đ., Fabek S., Toth N., Vorkapić-Furač J., Redovniković I.R. (2012). Optimisation of microwave-assisted extraction of phenolic compounds from broccoli and its antioxidant activity. Int. J. Food Sci. Technol..

[62] Z. Xu, Important antioxidant phytochemicals in agricultural food products, Analysis of antioxidant-rich phytochemicals, (2012) 1-24.

[63] Hsieh Y.-H., Li Y., Pan Z., Chen Z., Lu J., Yuan J., Zhu Z., Zhang J. (2020). Ultrasonication-assisted synthesis of alcohol-based deep eutectic solvents for extraction of active compounds from ginger. Ultrason. Sonochem..

[64] Kumar K., Srivastav S., Sharanagat V.S. (2021). Ultrasound assisted extraction (UAE) of bioactive compounds from fruit and vegetable processing by-products: a review. Ultrason. Sonochem..

[65] Zhang Q., Vigier K.D.O., Royer S., Jérôme F. (2012). Deep eutectic solvents: syntheses, properties and applications. Chem. Soc. Rev..

[66] Zhu S., Zhou J., Jia H., Zhang H. (2018). Liquid–liquid microextraction of synthetic pigments in beverages using a hydrophobic deep eutectic solvent. Food Chem..

[67] Alasalvar H., Yildirim Z. (2021). Ultrasound-assisted extraction of antioxidant phenolic compounds from Lavandula angustifolia flowers using natural deep eutectic solvents: an experimental design approach. Sustain. Chem. Pharm..

[68] Yin X.-S., Zhong Z.-F., Bian G.-L., Cheng X.-J., Li D.-Q. (2020). Ultra-rapid, enhanced and eco-friendly extraction of four main flavonoids from the seeds of Oroxylum indicum by deep eutectic solvents combined with tissue-smashing extraction. Food Chem..

[69] Bhargava N., Mor R.S., Kumar K., Sharanagat V.S. (2021). Advances in application of ultrasound in food processing: a review. Ultrason. Sonochem..

[70] Patil S.S., Pathak A., Rathod V.K. (2021). Optimization and kinetic study of ultrasound assisted deep eutectic solvent based extraction: a greener route for extraction of curcuminoids from *Curcuma longa*. Ultrason. Sonochem..

[71] Mjalli F.S., Ahmad O. (2017). Density of aqueous choline chloride-based ionic liquids analogues. Thermochim Acta.

[72] Naseem Z., Zahid M., Hanif M.A., Shahid M. (2020). Environmentally friendly extraction of bioactive compounds from Mentha arvensis using deep eutectic solvent as green extraction media. Pol. J. Environ. Stud..

[73] Vongsak B., Sithisarn P., Mangmool S., Thongpraditchote S., Wongkrajang Y., Gritsanapan W. (2013). Maximizing total phenolics, total flavonoids contents and antioxidant activity of *Moringa oleifera* leaf extract by the appropriate extraction method. Ind. Crop. Prod..

[74] Hilary S., Tomás-Barberán F.A., Martinez-Blazquez J.A., Kizhakkayil J., Souka U., Al-Hammadi S., Habib H., Ibrahim W., Platat C. (2020). Polyphenol characterisation of Phoenix dactylifera L. (date) seeds using HPLC-mass spectrometry and its bioaccessibility using simulated in-vitro digestion/Caco-2 culture model. Food Chem..

[75] Sirisena S., Zabaras D., Ng K., Ajlouni S. (2017). Characterization of date (Deglet Nour) seed free and bound polyphenols by high-performance liquid chromatography-mass spectrometry. J. Food Sci..

[76] Shen S., Wang J., Chen X., Liu T., Zhuo Q., Zhang S.-Q. (2019). Evaluation of cellular antioxidant components of honeys using UPLC-MS/MS and HPLC-FLD based on the quantitative composition-activity relationship. Food Chem..

