# Supplementary material for: Utilization of natural deep eutectic solvents and ultrasound-assisted extraction as green extraction technique for the recovery of bioactive compounds from date palm (Phoenix dactylifera L.) seeds: An investigation into optimization of process parameters
Source: Ultrason Sonochem. 2022 Nov 21;91:106233. doi: 10.1016/j.ultsonch.2022.106233 (PMC9703823; doi:10.1016/j.ultsonch.2022.106233)
Supplement: Supplementary Data 1 [file mmc1.docx]

# Supplementary Material

# S.1 Materials and Methods

S 1.1. **Determination of total phenolic content (TPC) and total flavonoid content (TFC)**

The TPC of the synthesized NADES were determined following the method described by (Olatunde et al., 2018) with slight modifications. Briefly, 20 μL of a known concentration of the date seed powder were pipetted in a 96-well microplate. To each well, 10% Folin-Ciocalteu reagent of 150 μL was pipetted and vortex. After 5 min, 150 μL of 6% Na_2_CO_3_ was added to the solution. The solutions were incubated for 1 h at 37 ℃ and the absorbance was taken at 760 nm in a spectrophotometer microplate reader (Multiskan Sky, Thermo Fisher Scientific, Cambridge, MA, USA). The reference standard concentrations ranges from was (0-200 µg/ml) gallic acid and the TPC was expressed as milligrams of gallic acid equivalents per gram of DSP (mg GAE g^-1^ DSP). The analysis was performed in triplicates.

Total flavonoid content (TFC) was estimated with the aluminium chloride assay described by ( Zhishen et al., 1999). An aliquot of the various extracts (30μL) was pipetted to the 96-wells. A 9μL of NaNO_2_ (5%) was added and after 5 min, 9 μL of AlCl_3_ (5%) was added. Then 60 μL NaOH (1 M) was pipetted to the solution. The solution was thoroughly mixed and incubated for 15 min, the absorbance was estimated at 510 nm, quercetin was used as standard. The results were expressed in mg quercetin equivalents per gram of DSP (mg QE g^-1^ powder).

**S 1.2 Determination of antioxidant activity**

The radical scavenging activity 1,1-diphenyl-2-picrylhydrazyl (DPPH) of the NADES-based date seed extracts was conducted as described by Mostafa et al., (2022). Briefly, 15 mM DPPH was prepared in methanolic solution, and 100 μL of the DPPH solution was added to 100 μL of the extract. The decrease in the absorbance was measured at 517 nm after 30 min with a spectrophotometer microplate reader (Multiskan Sky, Thermo Fisher Scientific, Cambridge, MA, USA). The results were expressed in mmol Trolox equivalents per gram of DSP (mmol TE/ g powder).

The ferric reducing antioxidant power (FRAP) assay was analyzed by the method of (Gullón et al., 2017). The freshly prepared working FRAP solution containing 2.5 mL of ferric chloride (20 mmol), 25 mL of sodium acetate buffer (300 mmol, pH = 3.6), and 2.5 mL of TPTZ (10 mmol in 40 mmol hydrochloric acid), incubated at 37 ℃ for 30 min. The 285 μL FRAP working solution was added to 15 μL of the extract. After 30 min, the absorbance was measured at 593 nm. The Trolox equivalents (mmol) were obtained using standard concentration of Trolox (0-800 µm/ml).

The ABTS scavenging assay was analyzed following the method described by Mostafa et al., (2022b) with slight modifications. The 5.2 mM potassium persulphate and 14.8 mM of 2, 2’-azino-bis (3-ethylbenzthiazoline-6-acid) (ABTS) solutions were prepared. ABTS●+ stock solution was prepared by measuring 1 ml of each solution and incubated in the dark for 12-16 hours. ABTS●+ working reagent was prepared by mixing 1 mL ABTS●+ stock solution with 60 mL methanol to obtain an absorbance of 0.8±0.02 units at 734 nm. Fresh ABTS●+ working solution was prepared for each assay, 15 µL extracts were mixed with 285 µL of the ABTS●+ solution and incubated for 2 h, then absorbance was measured at 734 nm. The standard concentration of trolox range from (0-600 µM/ml). The results were expressed in mmol Trolox equivalents per gram of DSP (mmol TE/ g powder).

**S 1.3 Identification and quantification of the major phenolic compounds in date seed powder**

A Thermo Scientific Dionex Ultimate 3000 UHPLC system equipped with a quaternary Series RS pump and a Thermo Scientific Dionex Ultimate 3000 Series TCC-3000RS column compartment with a Thermo Fisher Scientific Ultimate 3000 Series WPS-3000RS autosampler and a rapid separations PDA detector controlled by Chromeleon 7.2 Software (Thermo Fisher Scientific, Waltham, MA, USA and Dionex Softron GmbH subsidiary of Thermo Fisher Scientific, Bremen, Germany). The date seed powder extracted with ChCI-LA and conventional solvents were analyzed. An aliquot of each 200 μL was filtered through a 0.45 μm micropore membrane (PTFE, Waters, Milford, MA, USA) before it was injected into the UHPLC-PDA. Liquid chromatography was performed using a UHPLC C18 column (Suplecosil LC-18-DD 150 X 4.6 mm). The mobile phase constitutes 0.1% formic acid in water (eluent A) and 0.1% formic acid in methanol (eluent B). The gradient program was as follows: 0–0.1 min, B (15%); 0.1–7.1 min, B (65%); 7.1–7.9 min, B (95%); 7.9–8.0 min, B (95%); 8.0–10.0 min, B (15%). The flow rate in the mobile phase was constant at 1.0 mL/min, the column temperature was set at 30 °C, and the effluents were measured at 280 nm.
